# Facial emotional processing deficits in long-term HIV-suppressed patients

**DOI:** 10.7448/IAS.17.4.19664

**Published:** 2014-11-02

**Authors:** Alicia Gonzalez-Baeza, Ignacio Perez-Valero, Fernando Carvajal-Molina, Carmen Bayon, Marisa Montes-Ramirez, Jose Ignacio Bernardino, Jose R Arribas

**Affiliations:** 1 HIV Unit, IdiPAZ Hospital Universitario La Paz, Madrid, Spain; 2 Psicologia Biologica y de la Salud, Universidad Autonoma de Madrid, Madrid, Spain; 3 Psiquiatria, IdiPAZ Hospital Universitario La Paz, Madrid, Spain

## Abstract

**Introduction:**

Emotional processing is basic for social behaviour. We examine for the first time the facial emotion processing in long-term HIV-suppressed patients.

**Materials and Methods:**

Cross-sectional study comparing (ANOVA) six facial emotional processing tasks (two discrimination, two memory and two recognition) between HIV-suppressed patients (HIV+) on effective antiretroviral therapy (>2 years) and matched (age, gender) healthy controls (HCs). Accuracy in the recognition of basic emotions (neutral, happiness, sadness, anger and fear) in each recognition task was also compared (Mann–Whitney U test) between HIV+ and HCs. In the subset of HIV+, we evaluate which factors were associated with impaired recognition of basic emotions (accuracy below 50%) by multiple logistic regression analysis. Overall performance in all six emotional tasks were separately compared between neurocognitive impaired and non-impaired HIV+.

**Results:**

We included 107 HIV+, mainly Caucasian (89%) male (72%) with a mean age of 47.4 years, neurocognitively non-impaired (75.5%), and 40 HCs. Overall discrimination (p=0.38), memory (p=0.65) and recognition tasks (p=0.29) were similar in both groups. However, HIV+ had lower sadness recognition in both recognition tasks and lower sadness, anger and fear recognition in the facial affect selection task (Figure 1). Only estimated pre-morbid functioning (WAIS-III-R vocabulary subtest score) was significantly associated with sadness (1.99 [95% CI 1.18–3.58]; p=0.01) and anger recognition deficits (2.06 [95% CI 1.14–3.45]; p=0.015) in the facial affect selection task. In HIV+ individuals, neurocognitive impairment was associated with worse memory task results (p<0.01, d=0.88; p<0.01, d=1.48).

**Conclusions:**

We did not find difference in the overall emotion processing between HIV+ and HIV- individuals. However, we found particular recognition deficits in the entire HIV+ sample. Estimated pre-morbid functioning was associated with sadness and anger recognition deficits in the facial affect selection task. Neurocognitive impaired HIV+ had additional memory deficits. These recognition deficits might conduct to social difficulties.

**Figure 1:**
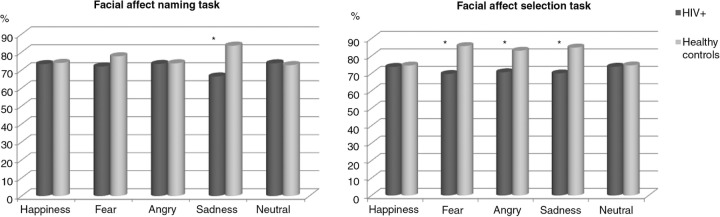
Percentage of correct recognition responses given in each specific emotion by HIV+ and healthy control participants. Note: Significant differences (p<.05) in distribution of correct response calculated by Mann-Whitney U-test. Axis Y=% of correct response.

